# How mindfulness and coping styles shape anxiety and depression in metastatic gastrointestinal cancer

**DOI:** 10.3389/fpsyg.2025.1659902

**Published:** 2025-09-16

**Authors:** Yasemin Bakkal Temi, Aila Gareayaghi, Ece Baydar, İlkay Çıtakkul, Devrim Çabuk, Umut Kefeli, Kazım Uygun

**Affiliations:** ^1^Department of Internal Medicine and Medical Oncology, Faculty of Medicine, Kocaeli University, Kocaeli, Türkiye; ^2^Department of Psychiatry, Faculty of Medicine, Kocaeli University, Kocaeli, Türkiye

**Keywords:** metastatic gastrointestinal cancer, palliative care, mindfulness, coping mechanisms, anxiety, depression, emotional regulation, psychological distress

## Abstract

**Objectives:**

Significant psychological distress is prevalent among patients with advanced gastrointestinal cancer, underscoring the need to investigate the roles of mindfulness and coping strategies in shaping emotional outcomes. However, this relationship has not yet been sufficiently explored. Therefore, this study aimed to investigate the associations between mindfulness, coping styles, and emotional distress (anxiety and depression) in individuals with advanced gastrointestinal cancer.

**Methods:**

This cross-sectional study enrolled 110 patients with stage IV gastrointestinal cancer who received palliative chemotherapy. Participants completed Turkish versions of the Mindful Attention Awareness Scale, Mental Adjustment to Cancer scale, and Hospital Anxiety and Depression Scale. Sociodemographic and clinical data were collected using a patient information form. Data were analyzed using non-parametric tests and correlation analyses to examine group differences and associations among study variables.

**Results:**

Our findings indicated significant associations between trait mindfulness, coping strategies, and emotional symptoms. Specifically, trait mindfulness was positively correlated with adaptive coping (*r* = 0.245, *p* = 0.010) and anxiety (*r* = 0.455, *p* < 0.001), whereas it was negatively correlated with maladaptive coping (*r* = −0.326, *p* = 0.001). Moreover, participants who employed maladaptive coping strategies reported lower levels of mindfulness. No significant differences in depression were observed between coping style groups. Notably, the positive correlation between adaptive coping strategies and anxiety symptoms highlights the complex nature of the coping mechanisms.

**Conclusion:**

These findings underscore the importance of contextualizing coping constructs, and suggest that mindfulness, despite its paradoxical relationship with anxiety, may mitigate reliance on maladaptive strategies. Routine psychological screening and personalized psychosocial support should be incorporated into oncological care to facilitate emotional adjustment.

## 1 Introduction

Gastrointestinal (GI) cancers represent a significant global health concern owing to their high incidence and mortality rates, collectively contributing to a substantial proportion of cancer-related deaths worldwide (Arnold et al., 2020; Wang et al., 2024). Despite advances in treatment modalities, including surgical intervention, systemic chemotherapy, radiotherapy, and in some cases, targeted or immunotherapy, achieving curative outcomes remains elusive in the context of metastatic disease (Koustas et al., 2022). Consequently, the primary objectives shift toward disease management, symptom alleviation, and extension of survival. Nevertheless, the impact of metastatic gastrointestinal cancer extends significantly beyond physical suffering, encompassing considerable psychological and social challenges (Cheng et al., 2024).

Advanced GI cancer exerts a significant psychological burden on patients, with elevated rates of anxiety and depression being documented within this population (Zamani and Alizadeh-Tabari, 2023). Meta-analytic evidence suggests that approximately one-fifth of patients with gastrointestinal cancer exhibit clinically significant anxiety symptoms, while nearly one-third experience depression (Zamani and Alizadeh-Tabari, 2023). These figures are often higher in cases of advanced-stage disease, in which patients encounter greater emotional and physical challenges. Individuals with limited social support or inadequate coping resources are particularly susceptible, and psychosocial stressors can exacerbate the emotional impact of cancer (Dev et al., 2023). Beyond personal suffering, this level of distress holds clinical significance: it is associated with diminished quality of life and may even interfere with medical care. Research has indicated that untreated depression or anxiety can negatively impact patients’ adherence to treatment and follow-up, resulting in suboptimal clinical outcomes such as reduced treatment completion and adherence (Cwik et al., 2021; Greer et al., 2008; Hansen et al., 2023). Furthermore, a recent meta-analysis encompassing over 60 cohort studies indicated that depression is significantly correlated with increased cancer-specific mortality across various cancer types, with an overall hazard ratio of 1.38 (Ungvari et al., 2025). Nonetheless, this association has not been consistently observed in gastrointestinal cancer.

In recent years, mindfulness – the nonjudgmental, present-focused awareness of internal and external experiences – has emerged as a key psychological factor that may buffer against emotional distress in patients with serious illnesses (Brown and Ryan, 2003; Cillessen et al., 2019; Heinen et al., 2024). Similarly, coping mechanisms, particularly those conceptualized by the Mental Adjustment to Cancer (MAC) framework, are known to influence psychological adjustment in cancer patients (Watson et al., 1988). Adaptive coping styles such as fighting spirit and acceptance have been linked to lower emotional distress, whereas maladaptive styles such as helplessness and anxious preoccupation are associated with increased depression and anxiety (Ghanem et al., 2020). Recent feasibility studies in gastrointestinal cancer cohorts have further substantiated the applicability of mindfulness-based interventions. Atreya et al. initially demonstrated the acceptability of an audio-based mindfulness program, “Being Present,” among patients with advanced gastrointestinal malignancies (Atreya et al., 2018). This initiative was subsequently expanded into a digital format, “Being Present 2.0,” which confirmed both feasibility and acceptability for patients and caregivers (Dragomanovich et al., 2021). More recently, the preliminary clinical outcomes presented at ASCO have further underscored its potential in advanced gastrointestinal cancers (Atreya et al., 2024), and an updated comprehensive review has emphasized the relevance of mindfulness-based interventions for integration into supportive care in gastrointestinal oncology (Atreya et al., 2025). Although previous research has explored mindfulness and coping mechanisms across various cancer populations, there is a paucity of studies specifically targeting patients with metastatic gastrointestinal cancer. This cohort was distinguished by poor prognosis, significant symptom burden, and unique psychological requirements. This study sought to fill this research gap by investigating the interrelationship between mindfulness, coping strategies, and emotional distress within this group.

To this end, we assessed these variables using three validated instruments: the Mindful Attention Awareness Scale (MAAS), Mental Adjustment to Cancer Scale (MAC), and Hospital Anxiety and Depression Scale (HADS). Our goal was to explore how mindfulness and coping styles relate to anxiety and depression in patients undergoing palliative chemotherapy for stage IV GI cancer. By doing so, we aimed to inform psychosocial care models tailored to the psychological realities of advanced cancer. Based on prior literature and international psycho-oncology guidelines (e.g., SIO/ASCO), we hypothesized that higher mindfulness would be associated with lower anxiety and depression symptoms. At the same time, we recognized that the psychological burden of advanced-stage disease could complicate this relationship, potentially producing both protective and adverse associations depending on illness stage, trauma history, and severity of distress. By addressing this complex interplay, our study sought to provide evidence that may inform tailored psychosocial care models for advanced cancer populations, and current clinical practice guidelines from the Society for Integrative Oncology and ASCO recommend mindfulness-based interventions to reduce anxiety and depression in cancer patients. This study adds a complementary perspective by highlighting the role of trait mindfulness. Assessing baseline mindfulness may help identify patients at a higher risk of distress and could inform personalized psychosocial care in future guideline updates. The significance of this research question lies in the fact that unmanaged psychological distress can adversely affect treatment adherence and quality of life within this vulnerable population. Consequently, elucidating the roles of mindfulness and coping strategies may inform the development of targeted psychosocial interventions.

## 2 Materials and methods

### 2.1 Patients

This descriptive cross-sectional study was conducted in the Department of Medical Oncology, Kocaeli University, between February and May 2025. All participants provided written informed consent prior to enrollment. The study protocol was approved by the Kocaeli University Clinical Research Ethics Committee (GOKAEK-2025/05/02) on January 30, 2025, and conducted in accordance with the Declaration of Helsinki. We thank the reviewer for this valuable comment. Patients were consecutively recruited from the outpatient clinic of the Department of Medical Oncology at Kocaeli University between February and May 2025. All eligible patients who met the inclusion criteria were approached in person by a research assistant during their routine clinic visits and invited to participate. No financial or other forms of compensation were provided for participation, and the study was conducted on a purely voluntary basis. We have clarified this point in the methods section of the revised manuscript.

The inclusion criteria were age ≥18 years, a histopathologically confirmed diagnosis of gastrointestinal cancer (gastric, colorectal, or pancreatic), having stage IV disease and receiving palliative chemotherapy within the first year of metastatic diagnosis, and voluntary agreement to participate. The exclusion criteria were early stage (I–III) disease, receipt of adjuvant chemotherapy, history of psychiatric illness, significant cognitive or physical impairments precluding informed consent, or refusal to participate.

The sample size required for this study was determined using the PASS 11 software, informed by previous data on factors influencing mental adjustment in cancer patients (Królikowska et al., 2024). With a 95% confidence level and 5% margin of error, the minimum sample size was calculated to be 105 participants. To mitigate the effects of potential attrition, 115 patients were recruited. However, five participants withdrew their consent, resulting in a final sample size of 110. In-person interviews were restricted to the administration of self-report scales and collection of sociodemographic information; no further qualitative data were gathered. The administered instruments included Turkish versions of the MAAS, MAC Scale, and HADS, all of which have been previously validated for use in Turkish populations (Karabekiroğlu et al., 2020; Çatak, 2012; Aydemir et al., 1997).

A structured patient information form was used to gather sociodemographic data. Residence was classified into three categories based on patient self-reports: urban/metropolitan areas (provincial centers and large cities), district towns, and rural/village settings. Additionally, it incorporated an open-ended question on the perceived objective of treatment. Responses were categorized as either “aware” (e.g., “to manage symptoms” or “to prolong life”) or “unaware” (e.g., “to be cured of cancer” or “I do not know”) based on predefined coding criteria. This classification was informed by prior research that underscored the significance of distinguishing between curative and palliative intent in patient comprehension (El-Jawahri et al., 2014).

### 2.2 Measurement instruments

The MAAS developed by Brown and Ryan (2003) was used to assess participants’ mindfulness levels. This unidimensional instrument evaluates individual differences in present-moment awareness and attention focus. It consists of 15 items rated on a 6-point Likert scale (1 = almost always to 6 = almost never), with total scores that are ranging from 15 to 90. Higher scores reflected greater mindful awareness. A Turkish validation study reported good internal consistency (Cronbach’s alpha = 0.80) (Çatak, 2012).

The MAC scale, developed by Watson et al., serves as a tool for evaluating patients’ cognitive and behavioral responses to cancer (Greer et al., 1989). This self-report instrument comprises 40 items, each rated on a 4-point Likert scale (1 = strongly disagree to 4 = strongly agree) that assess five distinct coping styles: Fighting Spirit, Avoidance, Helplessness/Hopelessness, Anxious Preoccupation, and Fatalism. In the context of Turkish validation, a bifactorial structure was adopted, categorizing the items into Positive Adjustment (21 items, encompassing Fighting Spirit and Avoidance) and Negative Adjustment (14 items, including the remaining three styles) (Karabekiroğlu et al., 2020). Elevated scores on Positive Adjustment are indicative of more adaptive coping mechanisms, whereas higher scores on Negative Adjustment suggest maladaptive tendencies. The score ranges were 21–84 for Positive Adjustment (with a cut-off of <56) and 14–54 for Negative Adjustment (with a cut-off of ≥36). The internal consistency of the scale is robust, with Cronbach’s alpha coefficients of 0.88 and 0.83 for the Positive and Negative subscales, respectively.

The HADS, originally developed by Zigmond and Snaith and subsequently validated in Turkish by Aydemir et al., was used to evaluate psychological distress (Cwik et al., 2021; Çatak, 2012). This self-report instrument consists of 14 items divided into two subscales, anxiety (HADS-A) and depression (HADS-D), each containing seven items rated on a 4-point Likert scale (0–3), resulting in subscale scores ranging from 0 to 21. In the Turkish adaptation, cut-off scores were established at ≥10 for anxiety and ≥7 for depression. Internal consistency was deemed acceptable, with Cronbach’s alpha coefficients of 0.85 for HADS-A and 0.77 for HADS-D (Aydemir et al., 1997). In the current study, the outcomes of the Hospital Anxiety and Depression Scale (HADS) were analyzed and reported solely as continuous variables to maintain methodological consistency.

### 2.3 Statistical analysis

All statistical analyses were performed using IBM SPSS for Windows version 29.0 (IBM Corp., Armonk, NY, United States). Kolmogorov–Smirnov and Shapiro–Wilk’s tests were used to assess the normality assumption. Continuous variables, including age, Mindful Attention Awareness Scale, MAC Positive Adaptation Scale, MAC Negative Adaptation Scale, HAD Anxiety Scale, and HAD Depression Scale, were summarized using the median and interquartile range (IQR), as the assumption of normality was not satisfied. Categorical variables, such as sex, marital status, occupation, educational level, and employment status, were presented as frequencies and percentages. Between-group comparisons of continuous variables were performed using the Mann–Whitney U test or Kruskal-Wallis test, as appropriate. When significant differences were identified using the Kruskal–Wallis test, Dunn’s test was applied for *post hoc* multiple comparisons. Associations between continuous variables were examined using Spearman’s correlation analysis. A *p*-value <0.05 was considered statistically significant.

## 3 Results

The median age of the participants was 64 years (IQR: 55–69 years), and the median duration since diagnosis was 8 months (IQR: 3–19 months). The median MAAS was 66 (IQR: 54.25–78), indicating a moderate level of trait mindfulness. Tests of normality indicated that variables such as age, trait mindfulness (MAAS), MAC Positive Adjustment, HADS-A, HADS-D, and illness duration deviated significantly from a normal distribution (all *p* < 0.05). By contrast, the MAC Negative Adjustment closely approximated a normal distribution (*p* = 0.066). Consequently, non-parametric statistical tests were performed Comprehensive sociodemographic and clinical characteristics of the sample are presented in Table 1.

**Table 1:** Sociodemographic and clinical characteristics of the study population.

Characteristic	*n* (%)
Sex	
Female	48 (43.6%)
Male	62 (56.4%)
Age, median (IQR)	64 (55–69)
Marital status	
Married	96 (87.3%)
Single/Divorced/Widowed	14 (12.7%)
Education level	
Primary	66 (60.0%)
High school	27 (24.5%)
University	17 (15.5%)
Occupation	
Retired	56 (50.9%)
Homemaker	33 (30.0%)
Worker	11 (10.0%)
Self-employed	8 (7.3%)
Officer	2 (1.8%)
Employment status	
Not working	89 (80.9%)
Working	21 (19.1%)
Residence	
City	38 (34.5%)
District	58 (52.7%)
Small Town/Village	14 (12.7%)
Cancer diagnosis	
Colon	78 (70.9%)
Gastric	25 (22.7%)
Pancreas	7 (6.4%)
Oncological treatment regimen	
FOLFOX-B	38 (34.5%)
FOLFOX-C/P	19 (17.3%)
FOLFİRİNOX	7 (6.4%)
FOLFOX	26 (23.6%)
FOLFİRİ-B	20 (18.2%)

*n*, number; %, percentage; IQR, interquartile range. Sample size: *n* = 110; FOLFOX-B, folinic acid, fluorouracil, oxaliplatin, and bevacizumab; FOLFOX-C/P, folinic acid, fluorouracil, oxaliplatin, and cetuximab/panitumumab; FOLFIRINOX, folinic acid, fluorouracil, irinotecan, and oxaliplatin; FOLFOX, folinic acid, fluorouracil, and oxaliplatin; FOLFIRI-B, folinic acid, fluorouracil, irinotecan, and bevacizumab.

The psychological and coping characteristics of the study population are detailed in Table 2. The median scores for mindfulness (MAAS), coping strategies (MAC positive and negative adjustment), and psychological outcomes (HADS-A and HADS-D) were presented as continuous variables, reflecting the overall distribution within the cohort.

**Table 2:** Psychological and coping characteristics of the study population.

Characteristic	Median (IQR)
MAAS (total score)	66 (54.25–78.0)
MAC positive adjustment	63.0 (58.0–71.0)
MAC negative adjustment	30.5 (25.0–35.0)
HADS-A	13.0 (10.75–15.0)
HADS-D	9.0 (7.0–11.0)

MAAS, Mindful Attention Awareness Scale; MAC, Mental Adjustment to Cancer; HADS-A, Hospital Anxiety and Depression Scale – Anxiety subscale; HADS-D, Hospital Anxiety and Depression Scale – Depression subscale.

No significant associations were identified between sociodemographic factors and mindfulness, coping strategies, or psychological outcomes. Consequently, anxiety (HADS-A) and depression (HADS-D) were analyzed and reported solely as continuous scores, ensuring alignment with the primary analytic framework.

Table 3 presents the socio-demographic and clinical correlates of mindfulness, coping strategies, and psychological outcomes. Although level of education demonstrated a marginal association with depression scores (*p* = 0.053), no significant associations were identified for variables such as sex, employment status, place of residence, primary cancer type, or treatment awareness.

**Table 3:** Sociodemographic and clinical factors associated with mindfulness, coping strategies, and anxiety/depression scores.

Sociodemographic/Clinical Factor	Mindfulness Total	MAC Positive Adjustment	MAC Negative Adjustment	HADS-A	HADS-D
Sex					
Female	65.0 (51.5–76.2)	66.0 (58.2–73.7)	32.0 (28.2–35.5)	12.0 (11.0–14.0)	9.0 (7.0–11.0)
Male	67.5 (54.2–79.0)	62.0 (58.0–68.5)	28.5 (24.7–35.0)	13.0 (10.0–15.0)	9.0 (8.0–11.2)
p^a^	0.361	0.088	0.073	0.254	0.354
Marital status					
Married	66 (56.2–77.7)	63.0 (58.0–70.0)	30.0 (25.0–35.0)	12.0 (10.2–14.7)	9.0 (8.0–11.0)
Not married	64.0 (49.7–79.5)	62.5 (58.7–78.0)	32.0 (27.0–41.2)	14.0 (12.2–15.0)	7.5 (5.7–11.0)
p^a^	0.918	0.243	0.304	0.304	0.097
Employment					
Working	65.0 (54.0–78.5)	60.0 (58.0–68.5)	31.0 (26.5–38.5)	13.0 (10.0–14.5)	10.0 (6.5–12.0)
Not working	66.0 (53.0–78.0)	64.0 (58.0–71.0)	30.0 (25.0–34.5)	13.0 (10.5–15.0)	9.0 (7.5–11.0)
p^a^	0.891	0.271	0.330	0.991	0.736
Education level					
Primary	65.0 (49.8–77.3)	64.5 (58.0–71.0)	31.0 (26.0–35.0)	12.0 (10.0–14.3)	9.0 (7.0–11.0)
High school	66.0 (52.0–76.0)	60.0 (57.0–69.0)	32.0 (26.0–42.0)	13.0 (10.0–14.0)	10.0 (8.0–12.0)
University	77.0 (63.0–80.5)	63.0 (58.0–71.0)	28.0 (25.0–31.5)	14.0 (13.0–15.5)	8.0 (6.0–11.0)
p^b^	0.129	0.933	0.246	0.014	0.053
Residence					
Urban city/metropolitan	66.5 (50.0–78.2)	62.0 (58.0–71.0)	29.5 (25.7–35.2)	13.0 (10.0–14.0)	8.0 (7.0–11.0)
District town	63.5 (55.7–77.2)	62.5 (57.7–70.0)	31.0 (25.7–35.0)	13.0 (10.0–15.0)	10.0 (9.0–11.2)
Rural (village)	73.0 (64.7–79.5)	66.5 (60.5–74.7)	32.0 (22.7–36.0)	12.5 (11.0–15.2)	9.0 (7.0–10.2)
p^b^	0.354	0.327	0.979	0.737	0.006
Diagnosis					
Colon cancer	65.0 (50.7–78.0)	63.5 (58.0–71.0)	31.0 (25.0–35.0)	13.0 (10.7–15.7)	9.0 (7.0–11.0)
Gastric cancer	66.0 (53.5–78.5)	62.0 (57.5–67.5)	29.0 (27.0–33.0)	13.0 (10.5–15.0)	9.0 (8.0–11.0)
Pancreas cancer	65.0 (58.0–78.0)	62.0 (55.0–73.0)	34.0 (27.0–41.0)	12.0 (10.0–13.0)	9.0 (7.0–11.0)
p^b^	0.922	0.353	0.739	0.611	0.697
Treatment awareness					
Accurate	65.0 (57.2–73.7)	62.5 (58.2–71.0)	29.0 (23.2–33.7)	13.0 (11.0–15.0)	9.0 (7.0–11.0)
Inaccurate	66.0 (50.0–79.0)	63.0 (58.0–70.0)	31.0 (27.0–37.0)	12.5 (10.0–14.2)	9.0 (8.0–11.0)
p^a^	0.622	0.467	0.095	0.904	0.671

P^a^: *p* values obtained by Mann–Whitney U test. P^b^: *p* values obtained by Kruskal–Wallis test. Data are presented as median (interquartile range). MAC, Mental Adjustment to Cancer; HADS, Hospital Anxiety and Depression Scale; MAC Positive, Positive coping strategies; MAC Negative, Negative coping strategies; HADS-A, Anxiety subscale; HADS-D, Depression subscale.

Spearman’s correlation analysis revealed that mindfulness scores were positively correlated with positive coping strategies (*r* = 0.245; *p* = 0.010) and HADS-A scores (*r* = 0.455; *p* < 0.001), while demonstrating a negative correlation with negative coping strategies (*r* = −0.326; *p* = 0.001). Table 4 displays the intercorrelation matrix of all the study variables. In addition to mindfulness, adaptive coping was positively correlated with anxiety (*r* = 0.263, *p* = 0.005), while maladaptive coping demonstrated a negative correlation with anxiety (*r* = −0.432, *p* < 0.001). No significant correlations were identified between depression scores, illness duration, and other constructs.

**Table 4:** Intercorrelation matrix of study variables.

Variables	Mindfulness total	MAC positive adjustment	MAC negative adjustment	HADS-A	HADS-D	Illness duration
Mindfulness total	1.000	0.245** (*p* = 0.010)	−0.326** (*p* = 0.001)	0.455** (*p* < 0.001)	−0.068 (*p* = 0.481)	0.066 (*p* = 0.492)
MAC positive adjustment	0.245** (*p* = 0.010)	1.000	−0.168 (*p* = 0.079)	0.263** (*p* = 0.005)	−0.167 (*p* = 0.080)	0.077 (*p* = 0.424)
MAC negative adjustment	−0.326** (*p* = 0.001)	−0.168 (*p* = 0.079)	1.000	−0.432** (*p* < 0.001)	0.114 (*p* = 0.237)	−0.005 (*p* = 0.955)
HADS-A	0.455** (*p* < 0.001)	0.263** (*p* = 0.005)	−0.432** (*p* < 0.001)	1.000	0.075 (*p* = 0.438)	−0.070 (*p* = 0.464)
HADS-D	−0.068 (*p* = 0.481)	−0.167 (*p* = 0.080)	0.114 (*p* = 0.237)	0.075 (*p* = 0.438)	1.000	0.055 (*p* = 0.567)
Illness duration	0.066 (*p* = 0.492)	0.077 (*p* = 0.424)	−0.005 (*p* = 0.955)	−0.070 (*p* = 0.464)	0.055 (*p* = 0.567)	1.000

Values indicate Spearman correlation coefficients (*r*) with corresponding *p*-values in parentheses. Correlations marked with ** are statistically significant at *p* < 0.01 (two-tailed). MAC, Mental Adjustment to Cancer; HADS, Hospital Anxiety and Depression Scale; MAC Positive, Positive coping strategies; MAC Negative, Negative coping strategies; HADS-A, Anxiety subscale; HADS-D, Depression subscale.

## 4 Discussion

This study is one of the few to investigate the interrelationships between mindfulness, coping strategies, and psychological distress, specifically anxiety and depression, in patients with advanced gastrointestinal cancer. Our findings indicated significantly heightened levels of psychological distress within the cohort, as evidenced by the elevated median scores for anxiety (HADS-A: 13.0) and depression (HADS-D: 9.0). The mindfulness scores were positively correlated with positive coping strategies and negatively correlated with negative coping strategies. Notably, patients who employed positive coping strategies demonstrated higher levels of mindfulness and anxiety. From a sociodemographic perspective, depression was more prevalent among individuals who were unmarried, had lower educational attainment, and resided in rural areas.

Mindfulness-based interventions have been explicitly endorsed in oncology guidelines as effective strategies to address psychological distress in cancer care. Recent joint guidelines from the Society for Integrative Oncology (SIO) and the American Society of Clinical Oncology (ASCO) strongly recommend meditation and mindfulness-based approaches for mitigating anxiety, depression, and mood disturbances in adults with cancer (Carlson et al., 2023). These recommendations are supported by evidence from diverse cancer populations; however, patients with advanced gastrointestinal malignancies remain significantly underrepresented in guideline synthesis. Our study contributes to expanding this evidence base by examining trait mindfulness rather than solely structured interventions, and by exploring its complex associations with coping strategies and sociodemographic factors in this high-burden population. In doing so, we provide complementary insights that may refine the implementation and tailoring of guideline-recommended practices to meet the unique psychological needs of patients with advanced gastrointestinal cancer.

In our study, depressive symptoms among patients with advanced gastrointestinal cancer were significantly correlated with various sociodemographic factors including employment status and residential setting. Depression scores varied according to employment status, with unemployed individuals exhibiting higher mean scores. Additionally, participants residing in district towns demonstrated elevated depression scores compared to those living in urban or metropolitan areas. These findings suggest that environmental stressors such as limited access to healthcare services, reduced social support, and economic hardship may contribute to increased psychological vulnerability. Supporting this interpretation, prior evidence indicates that non-metropolitan residences (district towns and rural villages) are associated with greater psychosocial distress, and financial difficulties exacerbate the psychological burden in cancer populations (Baye et al., 2023; Yu et al., 2022). For instance, inadequate social support increases the risk of depression, while unemployment and lower educational attainment are significant predictors of depressive symptoms among patients with cancer (Chen et al., 2024; Ikhile et al., 2024) and unemployment within the first 5 years following a cancer diagnosis has been significantly associated with elevated depression levels (Brink et al., 2024).

Notably, these sociodemographic characteristics did not exhibit a significant association with anxiety symptoms or coping style. This suggests that depression and anxiety may follow distinct psychosocial trajectories. Depression appears to be more closely associated with external and social factors, such as social isolation or occupational disruption, whereas anxiety may be more responsive to internal and clinical variables, including disease severity, symptom burden, and uncertainty regarding prognosis. Supporting this distinction, an umbrella review identified unemployment and inadequate social support as reliable predictors of depression but not anxiety (Getie et al., 2025). Conversely, anxiety is more frequently associated with advanced disease and physical deterioration (Tang et al., 2024; Teunissen et al., 2007). Furthermore, our data indicated that patients with higher educational levels had significantly higher anxiety scores. A previous study also observed that highly educated patients may possess greater health literacy and prognostic awareness, potentially exacerbating anticipatory anxiety in the absence of effective coping resources (Arvanitou et al., 2023). These findings highlight the necessity to implement tailored psychosocial interventions that consider educational levels and perceived illness insights within this population, potentially delivered through telehealth, telepsychiatry, or home-based services by nurses and social workers.

Interestingly, our study identified that elevated mindfulness scores were associated with increased anxiety symptoms, despite also showing a positive correlation with adaptive coping strategies. This counterintuitive finding may reflect the heightened emotional awareness that accompanies advanced mindfulness, whereby individuals become more attuned to distressing thoughts and emotions. Alternatively, it is plausible that individuals with preexisting anxiety are more inclined to adopt mindfulness practices as a compensatory coping mechanism, which could temporarily exacerbate emotional discomfort. Such bidirectional interpretations are supported by prior literature, in which mindfulness-based interventions (MBIs) have demonstrated variable short-term effects, particularly in advanced cancer populations (Rebegea et al., 2024; Oberoi et al., 2020). Importantly, however, longitudinal studies indicate that MBIs tend not to correlate with heightened anxiety after sustained practice (e.g., >6 months), suggesting that early elevations in emotional awareness may give way to longer-term psychological benefits. This distinction underscores the difference between cross-sectional assessments of “level of mindfulness” and the enduring effects of structured mindfulness training. Emerging research is also beginning to explore how much ongoing practice is required to maintain these benefits and achieve stable reductions in anxiety and depression. Taken together, these findings emphasize that mindfulness is not uniformly protective, and its effects are likely shaped by individual psychological context, stage of illness, and the integration of mindfulness practice within broader psychotherapeutic frameworks (Rebegea et al., 2024). Indeed, our results should be interpreted in light of the complex interplay between mindfulness and pre-existing psychological states. Patients with significant anxiety or prior traumatic experiences may have greater difficulty engaging in mindfulness, which could contribute to lower scores or even short-term exacerbations of distress. Moreover, because our study did not assess the amount or duration of actual mindfulness practice, the extent to which the observed associations reflect dispositional mindfulness versus structured training remains unclear. Future research should aim to disentangle these dynamics by employing more nuanced measures such as the Five Facet Mindfulness Questionnaire (FFMQ), which can capture distinct facets of mindfulness and clarify differential effects across patient subgroups.

The psychological impact of mindfulness may be affected by coping strategies used by patients. In our study, those who used positive adjustment strategies exhibited significantly higher mindfulness scores, supporting prior evidence that acceptance- and mindfulness-based interventions, such as Acceptance and Commitment Therapy (ACT), enhance psychological flexibility by fostering openness to negative emotions and reducing experiential avoidance (Oberoi et al., 2020; Jiang et al., 2023). Mindfulness, in turn, has been linked to more resilient emotional responses through improved metacognitive skills and internal regulation (Jiang et al., 2023; Zhang et al., 2022). However, a notable paradox emerged: higher “positiveadjustment” was also associated with greater anxiety. This finding likely reflects the inclusion of Avoidance within the positive adjustment subscale of the Turkish MAC scale. Although psychometrically grouped with adaptive styles in this version, avoidance often operates as a denial-based strategy that, while offering short-term relief, can impede emotional processing and ultimately heighten distress. Thus, while adaptive coping and mindfulness are generally aligned, the cultural framing of certain strategies such as avoidance complicates interpretation. These results underscore the need for caution when evaluating “positive adjustment” scores and highlight that the psychological benefits of mindfulness may vary depending on a patient’s broader coping repertoire, cultural context, and capacity for emotional insight (Czerw et al., 2016). These findings underscore that the effectiveness and psychological interpretation of mindfulness may vary depending on a patient’s broader coping repertoire and level of emotional insight.

The current findings emphasize the importance of incorporating psychological assessments and targeted interventions into standard oncology practices for patients with advanced gastrointestinal cancer. Specifically, routine screening and personalized psychosocial support programs may enhance treatment adherence and improve the quality of life among high-risk individuals, such as those who are unemployed, have lower educational attainment, or reside in rural areas. Furthermore, the paradoxical associations observed in patients with higher levels of mindfulness or positive coping, such as increased anxiety, highlight the complexity of emotional adaptation and suggest that certain psychological traits may not invariably confer protection. In this context, the implementation of individualized psycho-oncological strategies, including mindfulness-based models integrated with ACT, cognitive-behavioral approaches, and structured psychiatric support, is clinically relevant for enhancing emotional resilience and promoting treatment compliance.

This study had several limitations. First, its cross-sectional design precludes causal inference, and reliance on self-report instruments may have introduced a bias. Second, the sample was drawn from a single tertiary care center, which may restrict the generalizability of our results. Third, the Turkish version of the MAC group avoidance scale within positive adjustment potentially contributes to the paradoxical association between adaptive coping and higher anxiety. Moreover, multiple subgroup and correlational analyses were performed without formal correction, increasing the risk of Type I error; therefore, these results should be considered exploratory. Some subgroup analyses were further limited by small and uneven cell sizes, which reduced statistical power. Moreover, multivariate adjustments for potential confounders (e.g., age, sex, and education) were not systematically applied. Although exploratory regression suggested that the main findings remained robust, the limited sample size precluded comprehensive modeling. Finally, the study did not assess the amount of mindfulness practice undertaken by participants, which limits the interpretation of whether observed associations stem from dispositional traits or learned practices. Future studies should employ more comprehensive instruments, such as the FFMQ, to better capture the multidimensional nature of mindfulness, which indicates that the findings should be interpreted as preliminary and hypothesis-generating. Future studies using larger, more diverse samples and multivariate longitudinal designs are warranted to confirm and expand these results.

## 5 Future directions

Future research should utilize longitudinal and mixed-method approaches to comprehensively elucidate the causal and experiential dynamics of mindfulness, coping strategies, and psychological distress in individuals with advanced cancer. Furthermore, interventional trials focusing on high-risk groups, such as those exhibiting low acceptance or high anxiety, despite employing adaptive coping mechanisms, may contribute to the refinement of psychosocial care in oncology.

## 6 Conclusion

Patients with advanced gastrointestinal cancer experience significant psychological distress, characterized by elevated levels of anxiety and depression. This is particularly prevalent among individuals who are unmarried, unemployed, have a lower educational attainment, or reside in rural areas. In addition to these sociodemographic factors, maladaptive coping strategies and low acceptance of illness contribute substantially to emotional suffering. Notably, higher mindfulness was associated with increased self-reported anxiety, potentially indicating a heightened emotional awareness. These findings highlight the necessity for early psychological assessment and integration of evidence-based psychosocial interventions, such as CBT, ACT, and mindfulness-based approaches, into routine cancer care. Proactive multidisciplinary support can enhance psychological resilience, treatment adherence, and overall quality of life.

## Data Availability

The datasets presented in this article are not readily available because the dataset is not publicly available due to ethical restrictions, but it can be shared by the corresponding author upon reasonable academic request and with appropriate ethical approval if required. Requests to access the datasets should be directed to YBT yasemin.temi@kocaeli.edu.tr.
